# 3D Liquid Marble Microbioreactors Support *In Vitro* Maturation of Prepubertal Ovine Oocytes and Affect Expression of Oocyte-Specific Factors

**DOI:** 10.3390/biology10111101

**Published:** 2021-10-25

**Authors:** Daniela Bebbere, Stefano Mario Nieddu, Federica Ariu, Davide Piras, Sergio Ledda

**Affiliations:** 1 Department of Veterinary Medicine, University of Sassari, 07100 Sassari, Italy; nieddino@hotmail.it (S.M.N.); federica@uniss.it (F.A.); giodi@uniss.it (S.L.); 2 Cell Dynamics S.r.l., Via Viola Cielia 62/d, 40026 Imola, Italy; davide.piras@celldynamics.it

**Keywords:** low developmental competence, gene expression, 3D in vitro culture

## Abstract

**Simple Summary:**

Oocyte in vitro maturation has broad potential for generating embryos for research and for application of assisted reproductive technologies, such as in vitro embryo production. In human, the possibility to efficiently mature oocytes in vitro would solve the reproductive problems of patients with special diseases. Nevertheless, the developmental ability of in vitro matured oocytes is currently lower than those matured in vivo. Here, we used young sheep oocytes as model of low-quality gametes to show that a novel liquid marble 3D culture system is suitable to mature in vitro oocytes with reduced potential, improving the rates of in vitro embryo production. The present findings are useful for the optimization of in vitro maturation systems, and to improve the developmental potential of in vitro matured oocytes. Further applications should be considered also in other species, including human, to mature oocytes with intrinsic low quality.

**Abstract:**

In vitro oocyte maturation (IVM) is a well-established technique. Despite the high IVM rates obtained in most mammalian species, the developmental competence of IVM oocytes is suboptimal. The aim of this work was to evaluate the potential beneficial effects of a liquid marble microbioreactor (LM) as a 3D culture system to mature in vitro prepubertal ovine oocytes, as models of oocytes with intrinsic low competence. Cumulus–oocyte complexes of prepubertal sheep ovaries were in vitro matured in a LM system with hydrophobic fumed-silica-nanoparticles (LM group) or in standard conditions (4W control group). We evaluated: (a) maturation and (b) developmental rates following in vitro fertilization (IVF) and embryo culture; (c) expression of a panel of genes. LM and 4W groups showed similar IVM and IVF rates, while in vitro development to blastocyst stage approached significance (4W: 14.1% vs. LM: 28.3%; *p* = 0.066). The expression of *GDF9*, of enzymes involved in DNA methylation reprogramming and of the subcortical maternal complex was affected by the IVM system, while no difference was observed in terms of cell-stress-response. LM microbioreactors provide a suitable microenvironment to induce prepubertal sheep oocyte IVM and should be considered to enhance the developmental competence of oocytes with reduced potential also in other species, including humans.

## 1. Introduction

Oocyte in vitro maturation (IVM) is an assisted reproductive technology designed to obtain mature oocytes following culture of immature cumulus–oocyte complexes (COCs) collected from antral follicles. It is a well-established technique largely applied to in vitro embryo production in the livestock field. In human, IVM implementation has been more challenging, and outcomes remain highly variable [1]; in the past decade, substantial improvements led to a more frequent application in clinical practice, which is expected to further increase in the future [2].

Despite the high IVM rates obtained in most mammalian species (ranging 60–90%; [3], the developmental competence of in vitro matured oocytes is still suboptimal, as indicated by the relative low development to blastocyst stage and the poor viability to term after embryo transfer into recipients.

Two main factors lead to successful oocyte IVM: the system of IVM and the intrinsic quality of the oocyte. Several strategies were developed in different species to improve the IVM system, including formulation of specific maturation media [4], addition of growth factors and molecules [5,6], co-culture with different somatic cells [7] and modulating the length of in vitro culture [8,9]. Nevertheless, when oocytes with intrinsic reduced quality are matured in vitro, they have low chance to reach the MII stage and further developmental stages. Specific strategies to improve the developmental potential are therefore needed to support the maturation of such low competence gametes.

In recent years, studies on three-dimensional (3D) cell cultures have seen a wave of interest that led to the creation of accurate physiological and pathological models [10]. Specific 3D systems were also developed to support oocyte IVM in different species: alginate microbeads [11], agarose matrix [12] or glass scaffolds [13]. In 2016, we developed a microbioreactor using liquid marbles (LM) consisting of a polytetrafluoroethylene (PTFE) particle bed, as a novel system to support ovine oocyte IVM in small volumes [5]. LM microbioreactors were seen to provide a microenvironment capable of supporting oocyte IVM conducive to blastocyst development.

The aim of the present work is to evaluate potential beneficial effects of a liquid marble microbioreactor (LM) as a 3D culture system to mature in vitro oocytes with low developmental competence. For such purposes, oocytes of prepubertal sheep donors were selected as an appropriate model of reduced developmental potential due to their ability to undergo normal embryo development and produce viable offspring, albeit with lower rates [14]. To investigate the mechanisms potentially affected by the IVM system, we analyzed the expression a panel of genes involved in crucial aspects of oocyte biology.

## 2. Materials and Methods

All chemicals in this study were purchased from Sigma-Aldrich S.r.l. (Milan, Italy) unless stated otherwise.

### 2.1. Ethics Approval

The oocytes used for in vitro experiments in the present study were harvested from ovaries collected at a local slaughterhouse in Sardinia, Italy, which does not require ethics approval.

### 2.2. Source of Oocytes and In Vitro Maturation

Ovaries of adult (4–6 years old) and prepubertal (30–40 days old) Sarda sheep were collected from a local slaughterhouse in PBS solution (Dulbecco’s phosphate buffered saline) containing penicillin (100 μg mL^−1^) and streptomycin (100 μg mL^−1^) at 37 °C. Cumulus–oocytes complexes (COCs) were recovered by slashing in sterile Petri dishes containing dissection medium (20 mM Hepes-buffered TCM 199 supplemented with 0.1% (*w*/*v*) polyvinyl alcohol (PVA) and antibiotics). COCs with a uniform cytoplasm and several layers of unexpanded cumulus cells [15] were selected and randomly divided between two different IVM systems as outlined below.

#### 2.2.1. Control Group (4W)

Groups of 30~35 COCs were matured in 600 μL of TCM 199 supplemented with 10% (*v*/*v*) estrus sheep serum (OSS), 0.1 IU mL^−1^ FSH and 0.1 IU mL^−1^ LH (Pergonal, Serono, Italy), 8 mg/mL of pyruvate and 100 mM cysteamine (IVM medium). COCs were cultured in four-well Petri dishes (Nunclon; Nalge Nunc International, Roskilde, Denmark) covered with 300 μL preequilibrated mineral oil for 24 h under 5% CO_2_ in air at 38.5 °C.

#### 2.2.2. Liquid Marble Group (LM)

**Figure 1:**
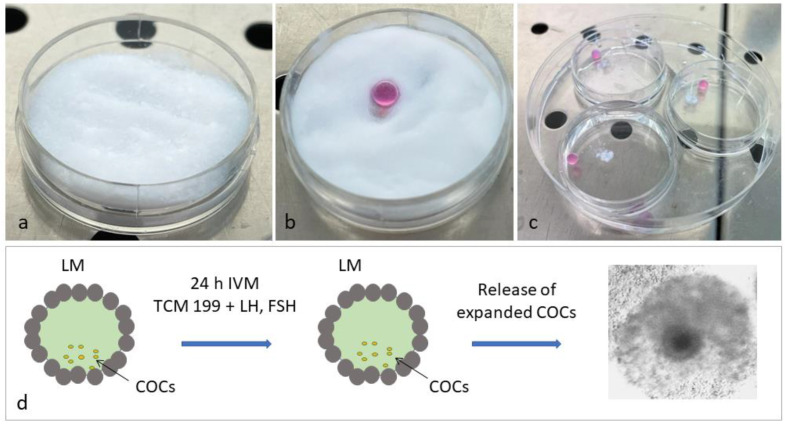
Preparation of liquid marbles (LM) containing COCs. (**a**) A hydrophobic Cab-O-Sil/Cabot powder bed is prepared in a 35-mm Petri dish. (**b**) Thirty μL of IVM medium, containing 10 COCs, are dispensed over the hydrophobic Cab-O-Sil/Cabot powder bed. The IVM drop is gently rolled over the powder to be fully coated with the Cab-O-Sil/Cabot particles. (**c**) The resulting LM drop is placed in a new 35-mm Petri dish positioned inside a larger Petri dish containing sterile water to prevent evaporation. (**d**) Schematic representation of IVM in Cab-O-Sil/Cabot powder LM.

The LM microbioreactor was created inside a Petri dish by preparing a hydrophobic fumed silica nanoparticle (Cab-O-Sil/Cabot) bed with particle size of 1 μm; a spatula was used to gently make a curved gully at the center of the powder bed (Figure 1). A micropipette was used to dispense the required volume (30 μL) of IVM medium, containing a predetermined number of COCs (10 COCs for each drop) on the Cab-O-Sil/Cabot bed. The Petri dish was then gently shaken in a circular motion to ensure that the powder particles completely covered the surface of the liquid drop. The LM drops were transferred to new 35-mm Petri dishes and incubated for 24 h at 38.5 °C in 5% CO_2_ in air. To increase humidity and avoid dehydration, the Petri dishes were placed in a larger Petri dish containing sterile water and all Petri dishes were capped. All experiments were performed in three replicates.

### 2.3. In Vitro Fertilization

After 24 h, in vitro matured prepubertal oocytes from the 4W and LM systems were subjected to in vitro fertilization (IVF). COCs from the LM drops were released by the addition of IVM culture volume (200 μL) over the LM drops. IVF was performed as previously described by Bebbere et al. [15], in synthetic oviductal fluid (SOF [16]) with 2% OSS, 1 μg mL^−1^ heparin, 1 μg mL^−1^ hypotaurine for 22 h at 38.5 °C and under a 5% CO_2_, 5% O_2_, and 90% N_2_ atmosphere in four-well Petri dishes with frozen/thawed spermatozoa selected by swim-up technique (1 × 10^6^ spermatozoa mL^−1^).

### 2.4. In Vitro Embryo Development

IVF presumptive zygotes were cultured for 8 days in SOF with essential and non-essential amino acids at oviductal concentration [17], 0.4% bovine serum albumin (BSA) under mineral oil, in four-well Petri dishes in maximum humidified atmosphere with 5% CO_2_, 5% O_2_ and 90% N_2_ at 38.5 °C. Cleavage rates were recorded 40–48 h after the beginning of in vitro fertilization. Blastocyst development was recorded on day 8 (day 0 = day of IVF).

### 2.5. Gene Expression Analysis

Gene expression analysis by real-time PCR was performed and is described according to MIQE guidelines [18] and in line with recent recommendations [19].

### 2.6. Sample Collection for Gene Expression Analysis

The RNA samples were isolated from pools of denuded oocytes derived from adult and prepubertal donors at the germinal vesicle (GV) stage and after IVM with the two systems (LM and 4W). Between 4 and 6 pools of 10 oocytes were analyzed per experimental group (Table 1). Oocytes were denuded via gentle pipetting to completely remove somatic cells and added to 30 μL RLT buffer (RNeasy Micro Kit, Qiagen, Hilden, Germany), snap-frozen in liquid nitrogen, and stored at −80 °C until RNA isolation.

### 2.7. RNA Isolation and Reverse Transcription

Total RNA was isolated from the groups of oocytes with the RNeasy Micro Kit (Qiagen, Hilden, Germany) following manufacturer’s instructions. Five picograms of luciferase mRNA (Promega, The Netherlands) were added to each group prior to RNA isolation to account for RNA loss during the isolation process. During the procedure, RNA was treated with DNase I to exclude any potential genomic DNA contamination. Isolated RNA was eluted in 12 μL RNase-free water and immediately used for reverse transcription polymerase chain reaction (RT-PCR). Reverse transcription was performed in a final volume of 20 μL, consisting of 75 mM KCl, 50 mM Tris- HCl (pH 8.3), 5 mM DTT, 3 mM MgCl_2_, 1 mM dNTPs, 2.5 μM random hexamer primers, 20 U RNase OUT and 100 U SuperScript III RT (all purchased at Invitrogen Corporation, Carlsbad, CA, USA). The reaction tubes were incubated at 25 °C for 10 min, at 42 °C for 1 h and at 70 °C for 15 min to inactivate the reaction. One tube without RNA and one with RNA, but without reverse transcriptase, were analyzed as negative controls. To quantify the mRNA recovery rate, 5 pg of luciferase mRNA (not subjected to RNA isolation) were subjected to cDNA synthesis as well.

### 2.8. Real-Time Polymerase Chain Reaction

Primers were designed with Primer3 software, 0.4.0 version (http://frodo.wi.mit.edu/primer3/ accessed on 2 February 2021). The selected amplified regions were all intron-spanning as a further precaution to prevent amplification of trace genomic DNA, in the unlikely event of incomplete DNA digestion by DNase I (Table 2). Relative transcript quantification was performed by real-time polymerase chain reaction (RT-PCR) in a Rotor-Gene Q MDx 5plex HRM (Qiagen). The PCR was performed in a 15 μL reaction volume containing 7.5 μL 2× Quantinova SYBR Green PCR Kit (Qiagen, Germany), 200 nM of each primer and cDNA equivalent to 0.25 oocytes. The PCR protocol consisted in two incubation steps (50 °C for 5 min and 95 °C for 2 min), followed by 40 cycles of amplification (95 °C for 15 s and gene-specific annealing temperature (see Table 2) for 30 s), a melting curve program (65–95 °C, starting fluorescence acquisition at 65 °C and measuring at 10-s intervals until the temperature reached 95 °C), and a cooling step to 4 °C. Fluorescence data were acquired during the annealing steps. To minimize handling variation, all samples were analyzed in the same run using a PCR master mix containing all reaction components apart from the sample. PCR products were analyzed by generating a melting curve to check specificity and identity of the amplification product.

The efficiency of PCR reaction for each primer pair was previously assessed by building a standard curve with serial dilutions of a known amount of template, covering at least 3 orders of magnitude, so that the calibration curve’s linear interval included the interval above and below the abundance of the targets. Only primers achieving an efficiency between 90 and 110% (3.6 > slope > 3.1) and a coefficient of correlation (r^2^) > 0.99 were used for the analysis. The size of the amplification products was confirmed by electrophoresis on a 2% agarose gel in TBE 0.5X stained with SYBR Safe (Invitrogen) and visualized by exposure to blue light. The PCR products were sequenced (Model 3130 xl Genetic Analyzer; Applied Biosystems, Foster City, CA, USA) after purification with MinElute PCR purification kit (Qiagen, Hilden, Germany), and sequence identity was confirmed with BLAST (http://www.ncbi.nlm.nih.gov/BLAST/ accessed on 10 April 2021). The relative quantification of all transcripts was performed after normalization against the number of oocytes and luciferase mRNA levels [20,21,22].

### 2.9. Statistical Analysis

Data were analyzed with GraphPad Prism version 8.0.0 for Windows (GraphPad Software, San Diego, CA, USA). In vitro maturation, fertilization and embryonic development rates were analyzed by chi-squared test at each time point. 

After testing for normality using the Kolmogorov–Smirnov test, gene expression data were analyzed with the general linear model analysis of variance (ANOVA), followed by Tukey’s post hoc comparison when significant differences among groups as a whole were observed. 

Differences were considered significant when *p* < 0.05.

## 3. Results

### 3.1. In Vitro Maturation and Development

After 24 h culture, the percentage of prepubertal COCs that reached MII did not differ between LM and 4W groups (88% vs. 92.1%, *p* = 0.374), and no statistical difference was observed in the fertilization rate either (LM 69.7% vs. 4W 78.1%, *p* = 0.248). Conversely, in vitro development to blastocyst stage showed a better performance after IVM in the LM system (LM: 28.26% vs. 4W: 14.06%) with a *p* value near the significance threshold (*p* = 0.066; Table 3).

### 3.2. Gene Expression Analysis

To investigate the dynamics of a panel of transcripts during oocyte maturation in the two systems (LM and 4W), their abundance was observed before and after maturation. In addition, the patterns of gene expression during IVM of prepubertal oocytes were compared with the patterns observed during maturation of adult oocytes, to identify potential features associated with the developmental potential. 

We selected genes involved in several crucial aspects of oocyte biology to gain an overall indication of oocyte reaction to the different environment (LM or 4W IVM system). We included: (i) components of the subcortical maternal complex, a recently discovered oocyte- and embryo-specific structure that affects several pathways ruling oocyte to embryo transition [23,24]; (ii) oocyte-secreted factors that play key roles in the oocyte developmental competence [25]; (iii) genes involved in cell stress response to address a potential response of the oocyte to a possibly adverse environment [26]; (iv) enzymes involved in DNA methylation reprogramming, which is fundamental for a proper epigenetic setup of the embryo and occurs immediately after fertilization, relying on the molecules stored in the gamete [27,28].

Specifically, the panel of analyzed genes comprised seven components of the subcortical maternal complex (*KHDC3*, *NLRP2*, *NLRP5*, *OOEP*, *PADI6*, *TLE6* and *ZBED3*), three genes involved in cell stress response (*BAX*, *HSP90b* and *SOD1*), genes encoding three oocyte-secreted factors (*BMP15*, *GDF9* and *YAP1*) and four enzymes involved in DNA methylation reprogramming in the early embryo (*DNMT1*, *DNMT3A*, *DNMT3B* and *TET3*). The presence of all analyzed transcripts was confirmed in all experimental groups.

### 3.3. Expression of the SCMC Components

**Figure 2:**
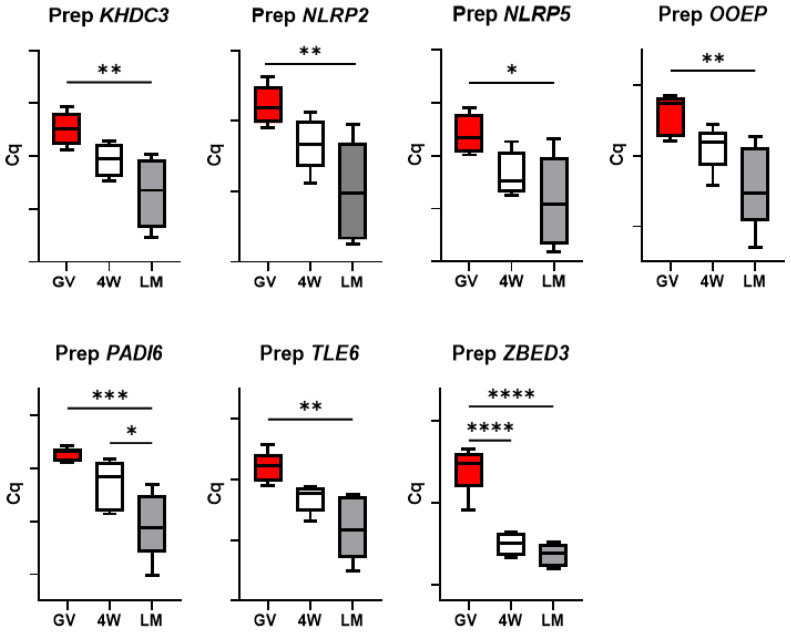
Expression of the subcortical maternal complex (SCMC) components in pools of prepubertal GV and MII oocytes matured in 4-well plates (4W) or in liquid marbles (LM). Target gene expression was normalized against the luciferase exogenous control. Relative abundance is expressed as ΔCq (*Y*-axis). Each box represents the mean expression of 4–6 replicate pools of 10 oocytes each (mean ± SE). Significant differences were assessed by ANOVA general linear model test; * *p* < 0.05, ** *p* < 0.01, *** *p* < 0.001, **** *p* < 0.0001.

In the prepubertal oocytes, a reduction in abundance of all transcripts was observed during maturation; such reduction is more prominent in the LM group (*p* < 0.05) compared to the 4W control (*p* > 0.05, except for *ZBED3* (*p* < 0.05 in both groups); Figure 2). 

**Figure 3:**
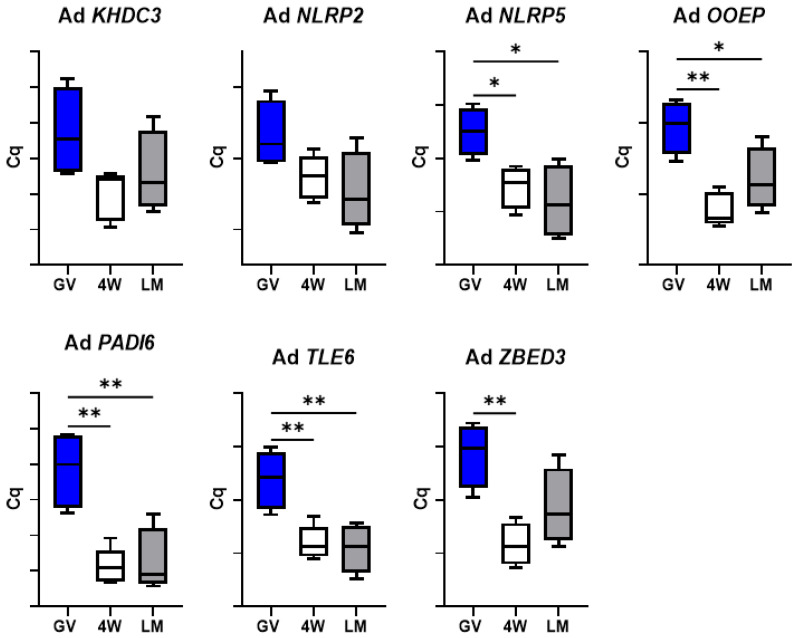
Expression of the subcortical maternal complex (SCMC) components in pools of adult GV and MII oocytes matured in 4-well plates (4W) or in liquid marbles (LM). Target gene expression was normalized against the luciferase exogenous control. Relative abundance is expressed as ΔCq (*Y*-axis). Each box represents the mean expression of 5 replicate pools of 10 oocytes each (mean ± SE). Significant differences were assessed by ANOVA general linear model test; * *p* < 0.05, ** *p* < 0.01.

Conversely, expression dynamics during maturation of adult gametes showed a similar trend in the two maturation systems for six genes (*KHDC3*, *NLRP2*, *NLRP5*, *OOEP*, *PADI6* and *TLE6*), while *ZBED3* decreases significantly in the 4W group, but not in the LM (Figure 3). More specifically, *KHDC3* and *NLRP2* mRNA abundance did not show any significant variation between GV, 4W and LM groups, while *NLRP5*, *OOEP*, *PADI6* and *TLE6* significantly decreased during maturation in both systems (*p* < 0.05).

### 3.4. Expression of Genes Involved in Cell Stress Response

**Figure 4:**
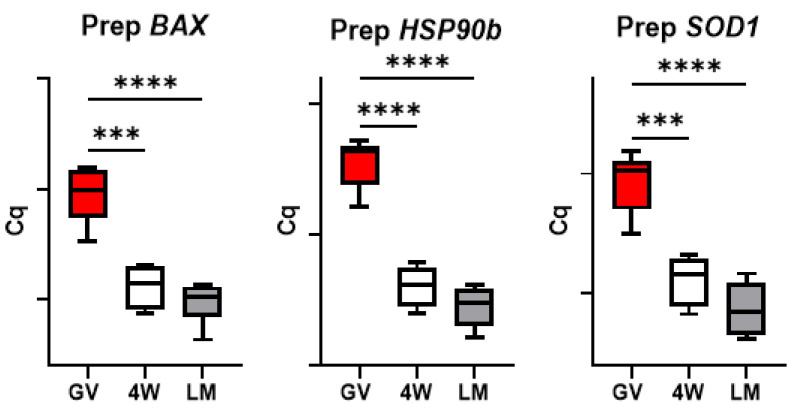
Expression of genes involved in cell stress response in pools of prepubertal GV and MII oocytes matured in 4-well plates (4W) or in liquid marbles (LM). Target gene expression was normalized against the luciferase exogenous control. Relative abundance is expressed as ΔCq (*Y*-axis). Each box represents the mean expression of 5 replicate pools of 10 oocytes each (mean ± SE). Significant differences were assessed by ANOVA general linear model test; *** *p* < 0.001, **** *p* < 0.0001.

**Figure 5:**
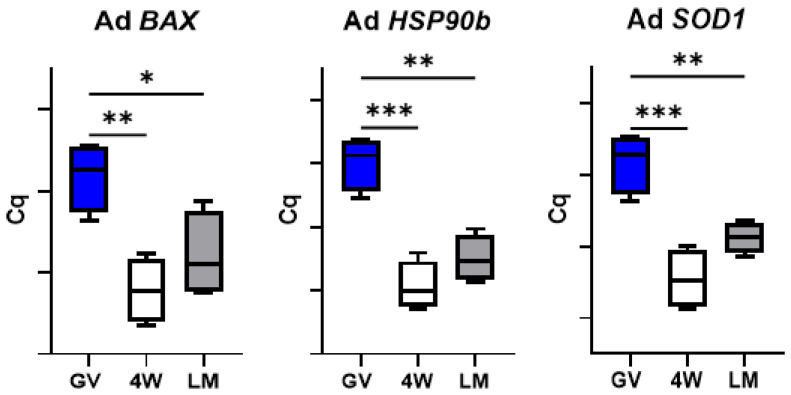
Expression of genes involved in cell stress response in pools of adult GV and MII oocytes matured in 4-well plates (4W) or in liquid marbles (LM). Target gene expression was normalized against the luciferase exogenous control. Relative abundance is expressed as ΔCq (*Y*-axis). Each box represents the mean expression of 5 replicate pools of 10 oocytes each (mean ± SE). Significant differences were assessed by ANOVA general linear model test; * *p* < 0.05, ** *p* < 0.01, *** *p* < 0.001.

A significant reduction in *HSP90b*, *SOD1* and *BAX* mRNAs abundance was observed during in vitro maturation of prepubertal and adult oocytes in both standard (4W) and LM systems (Figure 4 and Figure 5).

### 3.5. Expression of Genes Encoding Oocyte-Secreted Factors (OSF)

**Figure 6:**
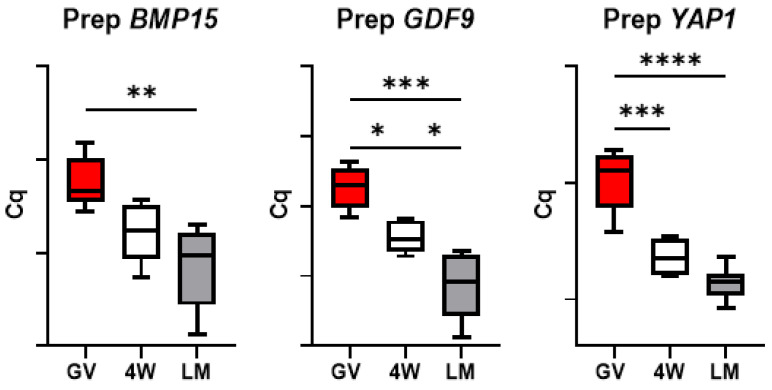
Expression of oocyte-secreted factors in pools of prepubertal GV and MII oocytes matured in 4-well plates (4W) or in liquid marbles (LM). Target gene expression was normalized against the luciferase exogenous control. Relative abundance is expressed as ΔCq (*Y*-axis). Each box represents the mean expression of 5 replicate pools of 10 oocytes each (mean ± SE). Significant differences were assessed by ANOVA general linear model test; * *p* < 0.05, ** *p* < 0.01, *** *p* < 0.001, **** *p* < 0.0001.

The oocyte secreted factor *BMP15* showed a significant reduction during maturation of prepubertal oocytes in the LM system, but not in the 4W (Figure 6). Conversely, *GDF9* transcript level decreased significantly in both 4W and LM systems but also showed a significant difference between the two MII groups (*p* < 0.05), with a more prominent reduction in the oocytes matured in LM (Figure 6). *YAP1* mRNA levels showed a significant drop over maturation in both IVM systems (*p* < 0.001).

**Figure 7:**
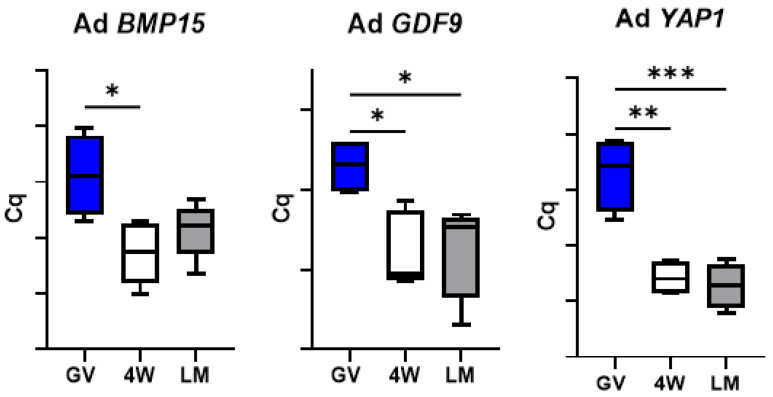
Expression of oocyte-secreted factors in pools of adult GV and MII oocytes matured in 4-well plates (4W) or in liquid marbles (LM). Target gene expression was normalized against the luciferase exogenous control. Relative abundance is expressed as ΔCq (*Y*-axis). Each box represents the mean expression of 5 replicate pools of 10 oocytes each (mean ± SE). Significant differences were assessed by ANOVA general linear model test; * *p* < 0.05, ** *p* < 0.01, *** *p* < 0.001.

Conversely, *GDF9* expression in adult oocytes dropped over maturation but did not show any difference between the two systems (Figure 7). Similarly, *YAP1* mRNA level was reduced after IVM in both system and showed no difference between 4W and LM (*p* < 0.01). Finally, *BMP15* transcript level decreased significantly during maturation in the standard system (4W; *p* < 0.05), but not in the LM. 

### 3.6. Expression of Enzymes Involved in DNA Methylation Reprogramming

**Figure 8:**
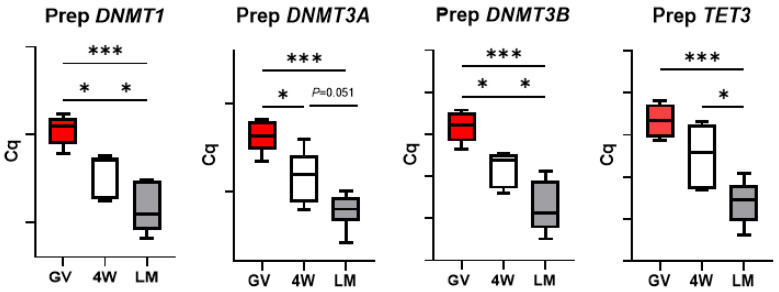
Expression of genes involved in DNA methylation reprogramming in pools of prepubertal GV and MII oocytes matured in 4-well plates (4W) or in liquid marbles (LM). Target gene expression was normalized against the luciferase exogenous control. Relative abundance is expressed as ΔCq (*Y*-axis). Each box represents the mean expression of 5 replicate pools of 10 oocytes each (mean ± SE). Significant differences were assessed by ANOVA general linear model test; * *p* < 0.05, *** *p* < 0.001.

Differently from the other groups of analyzed genes, the expression of the enzymes involved in DNA methylation reprogramming in prepubertal oocytes was always affected by the type of IVM system. *DNMT1*, *DNMT3A* and *DNMT3B* expression decreased after maturation in both systems, while *TET3* abundance did not vary after maturation in the standard 4W, but significantly dropped in the LM (*p* < 0.001; Figure 8). Importantly, *TET3*, *DNMT1*, *DNMT3A* and *DNMT3B* transcript level differed between 4W and LM groups, with a more prominent reduction in the oocytes matured in LM (Figure 8). 

**Figure 9:**
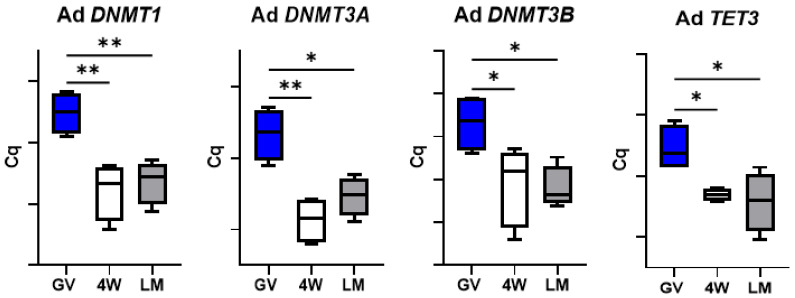
Expression of genes involved in DNA methylation reprogramming in pools of adult GV and MII oocytes matured in 4-well plates (4W) or in liquid marbles (LM). Target gene expression was normalized against the luciferase exogenous control. Relative abundance is expressed as ΔCq (*Y*-axis). Each box represents the mean expression of 5 replicate pools of 10 oocytes each (mean ± SE). Significant differences were assessed by ANOVA general linear model test; * *p* < 0.05, ** *p* < 0.01.

On the contrary, expression of the four genes in adult oocytes did not differ after IVM in the two systems (Figure 9).

## 4. Discussion

The present work showed that LM microbioreactors provide a suitable microenvironment to induce prepubertal oocyte IVM and may be used to enhance their developmental competence, as indicated by the improved blastocyst rates and by the expression patterns of specific genes. 

LM is a scaffold-free 3D microbioreactor that provides the most suitable conditions for cell aggregation. Allowing the maintenance of the physiological architecture of female gametes, it better simulates the follicular environment during meiotic maturation. In accordance, we previously showed that LM properly supports IVM of ovine oocytes of adult donors, with similar developmental rates to standard IVM systems [5]. In the present work, we show that oocytes with reduced developmental competence further benefit from a 3D culture system that minimizes the negative effects of the traditional 2D IVC (i.e., cumulus cell flattening and adhesion to the 2D support). COCs suspension in the maturation medium preserves physiological cell organization, with potential positive effects on oocyte and cumulus cell molecular, metabolic and developmental competence [29].

The blastocyst rate observed for prepubertal oocytes cultured in the LM microbioreactors is higher compared to the rate observed for the standard IVM system, with a relative *p* value near the significance threshold (*p* = 0.066). Possibly, the blastocyst rate did not reach statistical significance owing to the small number of IVP embryos (Table 3).

To gain further insights on the effects of IVM in LM, we considered the gene expression status of the oocyte before maturation and after IVM in the LM and in the standard (4W) systems. The oocyte transcriptome is assembled during the growth phase of oogenesis and is completed with the arrest of transcription in full-grown prophase I GV oocytes [30]. Oocyte maturation is associated with a major wave of maternal RNA degradation [22]. This absolute reduction in mRNA content, due to translation or active degradation, results in relative changes to transcript dosage in the MII transcriptome [22,31], which are fundamental for the establishment of oocyte competence to sustain fertilization and early embryo development [32,33]. 

Presuming a potential effect of the in vitro system on transcript use (translation or degradation), we evaluated the expression of a panel of genes involved in important processes of oocyte maturation and early embryo development. We selected seven components of the subcortical maternal complex (SCMC), three genes involved in cell stress response, three genes encoding oocyte-secreted factors and four encoding enzymes involved in DNA methylation reprogramming in the early embryo. This last group of genes was the most affected by the IVM system. The genes encoding three DNA methyl transferases (*DNMT1*, *DNMT3A* and *DNMT3B*) and the enzymes *TET3*, which is responsible for DNA hydroxymethylation leading to demethylation [27], all showed a significantly lower transcript abundance in the oocytes matured in LM (Figure 8). This suggests a more efficient use of the mRNAs in the 3D system. *DNMT* and TET enzymes regulate the genome-wide DNA methylation reprogramming that occurs in early embryos, immediately after fertilization [27,28]; such epigenetic remodeling is essential for the new developmental program of the nascent embryo and is therefore crucial to oocyte developmental competence. 

Oocyte-secreted factors (OSFs) are further key players in the acquisition of oocyte developmental competence, being involved in a bidirectional cross-talk via gap junctions between oocyte and cumulus cells [25]. Among them, *GDF9* and *BMP15* are crucial regulators of the growth and differentiation of granulosa cells, which in turn supply the oocyte with the support necessary for future healthy embryo/fetal development [25]. The different expression of *GDF9* we observed after maturation in the two systems suggests that LMs better preserve the physiological communication between somatic cells and the oocyte compared to the standard 4W system (Figure 6). Three dimensional in vitro culture probably allows better maintenance of COCs architecture and communication through gap-junctions and ensures proper functional activities.

Even if not different in terms of transcript abundance between LM and 4W, some genes showed diverse patterns of mRNA regulation over transcription, with a more prominent decrease over maturation in the LM system (*p* < 0.05) and a less evident reduction in the standard IVM conditions (4W; *p* < 0.05). This was observed in six of the seven components of the SCMC (Figure 2) and in one OSF (*BMP15*; Figure 6) and indicates by some means an effect of the IVM system on these transcripts turnover. The SCMC is a crucial structure for oocyte biology, being involved in several key processes leading the transition from oocyte to embryo, including meiotic spindle formation and positioning, regulation of translation, organelle redistribution, and epigenetic reprogramming [23,24]. The complex is also involved in human genetic infertility, therefore any variation in its expression most probably affects the oocyte developmental potential.

Finally, two further aspects deserve attention. The similar transcript abundance of genes involved in cell stress response in the two IVM systems (Figure 4) indicates the absence of a specific stress due to the LM system itself. In addition, the significant differences observed between the two systems (LM and 4W) in the prepubertal oocytes were not found in the adult counterpart. This was observed for the genes involved in DNA methylation reprogramming (Figure 9), for *GDF9* (Figure 7) and for the SCMC components (except *ZBED3*; Figure 3) and is in accordance with the in vitro developmental rates (Table 3). In our previous work, we indeed observed similar developmental rates for adult oocytes matured in LM or in standard conditions [5], while in the present work we report a better performance of prepubertal oocytes in the LMs.

## 5. Conclusions

In conclusion, LM microbioreactors provide a suitable microenvironment to induce oocyte IVM and may be used to improve the developmental competence of prepubertal ovine oocytes. Such system should be considered to improve the maturation and developmental rates of gametes with intrinsic low developmental competence also in other species. In human clinics, improving the success rate by optimizing the IVM culture system has a major significance. Many human oocytes exhibit maturation arrest in vitro because of intrinsic low developmental competence due to several factors (age, genetics, pathologies, exposure to environmental or behavioral conditions). Therefore, identifying novel IVM systems specifically beneficial to low competence oocytes is significant to the improvement of assisted reproductive technologies.

## Figures and Tables

**Table 1:** Number of pools of ten oocytes used for gene expression analysis.

	GV	LM—MII	4W—MII
Adult	4	5	5
Prepubertal	5	6	5

**Table 2:** Primers used for real-time PCR experiments. Ta: annealing temperature. Bps: base pairs.

Symbol	Gene Name	Accession Number	Primer Sequence	Ta	bps
*BAX*	BCL2 associated X protein	XM_004015363	5′ ctccccgagaggtctttttc 3′ 5′ tcgaaggaagtccaatgtcc 3′	58 °C	176
*BMP15*	Bone morphogenetic protein 15	NM_001114767	5′ gggttctacgactccgcttc 3′ 5′ ggttactttcaggcccatcat 3′	59 °C	173
*DNMT1*	DNA methylation transferase 1	NM_001009473	5′ cagctctcgtacatccacag 3′ 5′ aatctcgcgtagtcttggtc 3′	60 °C	158
*DNMT3A*	DNA methylation transferase 3A	XM_015094252	5′ gtgatgattgatgccaaaga 3′ 5′ ggtcctcactttgctgaact 3′	60 °C	165
*DNMT3B*	DNA methylation transferase 3B	XM_012189044	5′ attgcaacagggtacttggt 3′ 5′ atatttgatgttgccctcgt 3′	60 °C	122
*GDF9*	Growth differentiation factor 9	NM_001142888	5′ cagacgccacctctacaaca 3′ 5′caggaaagggaaaagaaatgg 3′	58 °C	198
*HSP90b*	Heat shock protein 90b	XM_004018854	5′ tggagatcaaccctgacca 3′ 5′ gggatcctcaagcgagaag 3′	58 °C	143
*KHDC3L*	KH domain containing 3 like	XM_027973471	5′ cagaccctgcttcacgttca 3′ 5′ cttctcagagcttcgcgcc 3′	60 °C	150
*LUC*	Luciferase reporter vector pXP2 *SA *PS	AF093685	5′ gctgggcgttaatcagagag 3′ 5′ gtgttcgtcttcgtcccagt 3′	58 °C	151
*NLRP2*	NLR family pyrin domain containing 2	XM_027977986	5′ gcatgtgttgctcattctgg 3′ 5′ agcactgtggaaacttgcag 3′	60 °C	120
*NLRP5*	NLR family pyrin domain containing 5	XM_027978862	5′ cagcctccaggagttctttg 3′ 5′ gacagcctaggagggtttcc 3′	59 °C	212
*OOEP1*	Oocyte expressed protein isoform 1	KF218578	5′ atccgctggtgttcttcctg 3′ 5′ gaacacggtgacttcgacc 3′	60 °C	149
*PADI6*	Peptidyl arginine deiminase, type VI	XM_012153966	5′ acggctgtactccacctcac 3′ 5′ cccagacccaggttctctta 3′	60 °C	109
*SOD1*	Superoxide dismutase 1	NM_001145185	5′caactcccgccagcagat 3′ 5′ ccgggaatggacagtcaca 3′	58 °C	130
*TET3*	Ten-eleven translocation 3	XM_015094461	5′tggagcatgtacttcaatgg 3′ 5′ ggtcacctggttctgatagg 3′	60 °C	173
*TLE6*	TLE family member 6	XM_004009373	5′ gctgcaggtctccatcatct 3′ 5′ ggatcagctcaagcagcatt 3′	60 °C	134
*YAP1*	Yes associated protein 1	XM_015100723	5′ ttcctttgagatccctgacg 3′ 5′ gtcctgccaggttgttgtct 3′	60 °C	115
*ZBED3*	Zinc finger BED-type containing 3	XM_027971476	5′ cccagggtagagtgtgcatt 3′ 5′ ggcaagggctactcatcaaa 3′	60 °C	97

**Table 3:** In vitro maturation, fertilization and development to blastocyst stage of prepubertal sheep oocytes cultured in liquid marble (LM) microbioreactor and control (4W) systems. * *p* = 0.066, chi-squared test.

	GV Oocytes	Matured Oocytes	Cleaved Embryos	Blastocysts
4W	89	82 (92.13%)	64 (78.13%)	9 (14.06%) *
LM	75	66 (88%)	46 (69.7%)	13 (28.26%) *

## Data Availability

The data produced during the current study are available from the corresponding author on reasonable request.
